# Sustained hole inversion layer in a wide-bandgap metal-oxide semiconductor with enhanced tunnel current

**DOI:** 10.1038/ncomms10632

**Published:** 2016-02-04

**Authors:** Gem Shoute, Amir Afshar, Triratna Muneshwar, Kenneth Cadien, Douglas Barlage

**Affiliations:** 1 Department of Electrical and Computer Engineering, University of Alberta, Edmonton, Alberta, Canada T6G 1H9; 2 Department of Chemical and Material Engineering, University of Alberta, Edmonton, Alberta, Canada T6G 1H9

## Abstract

Wide-bandgap, metal-oxide thin-film transistors have been limited to low-power, n-type electronic applications because of the unipolar nature of these devices. Variations from the n-type field-effect transistor architecture have not been widely investigated as a result of the lack of available p-type wide-bandgap inorganic semiconductors. Here, we present a wide-bandgap metal-oxide n-type semiconductor that is able to sustain a strong p-type inversion layer using a high-dielectric-constant barrier dielectric when sourced with a heterogeneous p-type material. A demonstration of the utility of the inversion layer was also investigated and utilized as the controlling element in a unique tunnelling junction transistor. The resulting electrical performance of this prototype device exhibited among the highest reported current, power and transconductance densities. Further utilization of the p-type inversion layer is critical to unlocking the previously unexplored capability of metal-oxide thin-film transistors, such applications with next-generation display switches, sensors, radio frequency circuits and power converters.

## Abstract (web summary)

Wide-bandgap, metal-oxide thin-film transistors are limited in their application by the lack of available p-type material. Here, the authors demonstrate a wide-bandgap metal-oxide n-type semiconductor that sustains a strong p-type inversion layer using a barrier dielectric with a high-dielectric constant.

## Graphical abstract

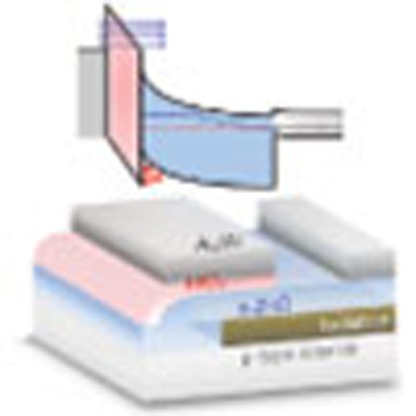

Metal-oxide thin-film transistors (TFTs) have shown increasing utilization in a wide range of applications. However, without a p-type inversion layer or controllable p-type doping mechanism^1^,^2^,^3^,^4^,^5^,^6^,^7^,^8^,^9^, these accumulation-mode transistors fail to exhibit the scalability of traditional metal-oxide semiconductor field-effect transistors or the versatility of silicon devices and consequently depend heavily on mobility enhancements to iteratively increase its performance^10^,^11^,^12^. For these reasons, TFTs have been limited to a niche application of low-power and low-frequency electronics such as display technologies. An inversion layer in wide-band gap n-type semiconductors is a fundamental challenge due to a low intrinsic carrier concentration correlated with its large band gap.

In the following, to realize this enabling component, a method to produce a two-dimensional inversion p-type layer induced in a wide-bandgap n-type thin-film is described. A heterogeneous p-type material is used to create a pn-junction with the active n-type semiconductor in order to supply the inversion layer at the metal-oxide semiconductor (MOS) interface. The detection of a hole inversion layer is carried out through a capacitance–voltage measurement. It is also compared with a MOS structure which has no supply of holes to inject to the interface. Further characterization of this structure, which includes an ultrathin high-κ dielectric, reveals the unexpected presence of enhanced tunnel current of electrons from the metal, through the barrier, into the wide-bandgap semiconductor. By taking advantage of the high current exhibited at this interface, a tunnelling-based TFT architecture is proposed and its realization yields a significant improvement of performance by measure of current, power and transconductance densities.

## Results

### Constituent materials of the MOS capacitor

**Figure 1:**
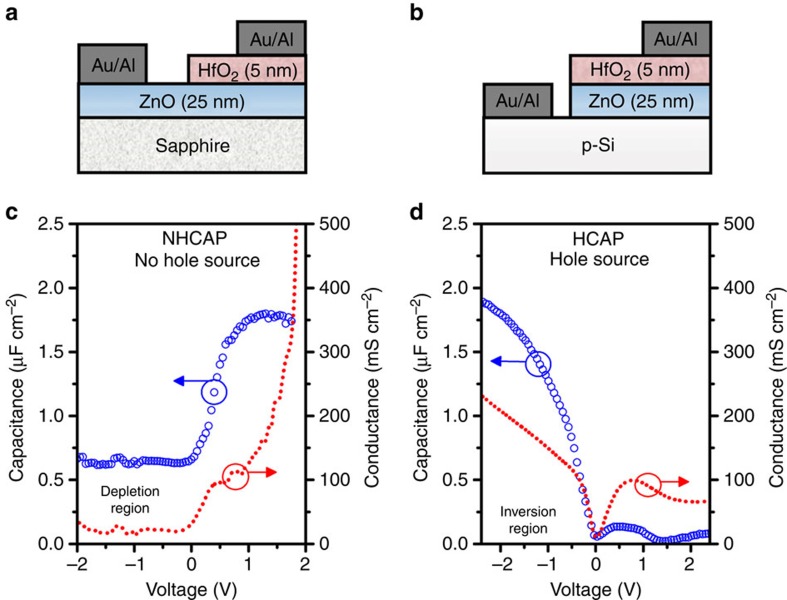
Capacitance–voltage characteristics. (**a**) The cross-section of the control MOSCAP HfO_2_/n-ZnO on a sapphire substrate (NHCAP) and (**b**) the hole source HfO_2_/n-ZnO/p-Si (HCAP). The capacitance and conductance measurements of (**c**) NHCAP revealing only an accumulation capacitance; and (**d**) HCAP revealing both inversion and accumulation capacitances. Furthermore, conductance curve for the HCAP case reveals that control over charge is maintained from *V*<0 V, but is minimal and eventual lost at *V*>1.5 V. For the NHCAP, charge is only controlled at the *V*>0 V.

In principle, any metallic oxide and wide-bandgap semiconductor could be used to realize this design. Because of its ease of fabrication and its multiple potential applications, zinc oxide (ZnO), an intrinsically n-type semiconductor whose properties are well known, was chosen^13^,^14^. An ultrathin-ZnO film (25 nm) was deposited on a p-type (100) silicon (p-Si) wafer. A thin (4 nm) high-κ oxide, HfO_2_, was employed to allow for the generation of a sufficiently large electric field at the gate to attract holes from an extrinsic source of p-type carriers at the oxide-semiconductor junction. A patterned metal layer (aluminium–gold) completed the fabrication process. A fabricated two-terminal MOS capacitor (MOSCAP) structure is shown in Fig. 1a clearly indicating the gate electrode. We expected the HfO_2_/n-ZnO/p-Si or ‘hole source' MOSCAP to operate primarily at inversion, because the p-Si would inject its majority hole carriers into n-ZnO. Also critical to this function, the n-ZnO film thickness was designed to be significantly lesser than its hole diffusion length^15^. A HfO_2_/n-ZnO control (null hypothesis) or ‘no holes source' MOSCAP was also fabricated consisting of just HfO_2_/n-ZnO grown on sapphire for comparison (Fig. 1b).

### Electrical characteristics of the MOS interface

The oxide-semiconductor interface was first electrically characterized using capacitance–voltage measurements for both the hole source (HCAP) and no hole source (NHCAP) MOSCAPs. In the NHCAP shown in Fig. 1c, there was a clear absence of an inversion layer at negative biases, along with the expected formation of an electron accumulation layer in ZnO at positively applied biases. The accumulation layer reached a large oxide capacitance density *C*_OX_ of 1.8 μF cm^−2^, which is significantly higher than its negative voltage counterpart which corresponded to the NHCAP's depletion region. In contrast, in Fig. 1d an inversion layer noticeably formed with the inclusion of a p-Si in the HCAP. The capacitance density *C*_OX_ reached as high as 1.9 μF cm^−2^ at a negative applied voltage corresponding to a p-type inversion layer concentration upwards of 10^20^ cm^−3^. The maximum capacitance in HCAP was greater than for the NHCAP implying that the p-type inversion layer was thinner than the n-type accumulation layer. This observation is in agreement with the larger charge centroid expected from the hole effective mass^16^. An accumulation capacitance of 0.12 μF cm^−2^ at positive voltages was also observed in the HCAP, this relatively miniscule capacitance arises from the depletion region in the reverse biased ZnO/p-Si junction.

The conductance–voltage was also measured for both cases since conductance has a direct relation with the amount of charge at the surface. For positive charge (holes), conductance would be dependent on negative applied voltages and vice versa for negative charge (electrons). As expected, in Fig. 1c a characteristic conductance–voltage was obtained for the HCAP where charge control was present at certain ranges for positive and negative voltages. In contrast, in Fig. 1d the mobile charge in the NHCAP structure was exclusively dependent on voltages at the positive range. However, for the HCAP, although charge control is maintained throughout the inversion voltage range, it was eventually lost at accumulation voltages greater than +1.5 V. This was consistent with the expected accumulation capacitance, which was considerably diminished because of a large ∼80 nm depletion region formed at the pn-junction on the p-Si side. These phenomena was not observed in the NHCAP device since the capacitance corresponded to the depletion thickness of the n-ZnO film alone.

### Enhanced tunnel current through an ultrathin barrier

**Figure 2:**
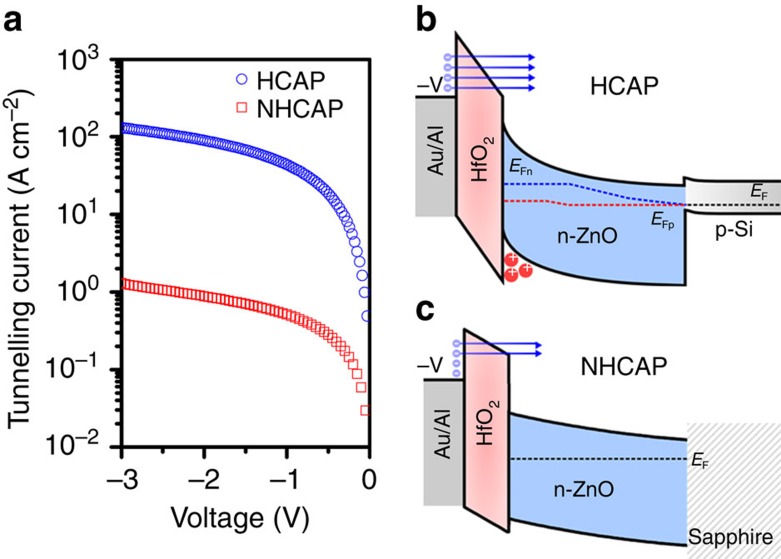
Tunnel current density at MOS interface. (**a**) The field-induced tunnelling current modulation at the tunnelling HfO_2_/n-ZnO interface for both NHCAP and HCAP. The band diagram for the: (**b**) HCAP, the injection of holes into wide-bandgap film caused a separation in the quasi-Fermi level of the two carrier gases (blue dotted line is the electron quasi-Fermi level and red dotted line is the hole quasi-Fermi level) and as a result, direct tunnelling from the metal electrode into the n-ZnO conduction band was enhanced; and (**c**) NHCAP where the n-ZnO is depleted at reverse biases and therefore minimizing tunnelling.

Current–voltage properties for both MOSCAPs through the 4 nm oxide at inversion voltages were also investigated. In Fig. 2a, a substantial enhancement of tunnelling current was detected with HCAP operating at inversion at the MOS interface which was not observed in the NHCAP. Comparatively, the tunnelling current of HCAP was greater by two orders. The band diagrams for the HCAP and NHCAP structures are given in Fig. 2b,c, respectively. In the NHCAP, the net current was limited by the depletion region formed at reverse bias. Distinct from the control structure, in the HCAP n-ZnO film, the quasi-Fermi level of the electrons (*E*_Fn_) and holes (*E*_Fp_) separate beginning from the n-ZnO/p-Si junction. This was a result of the injection of holes into the depleted n-ZnO from p-Si, effectively increasing the allowed states for tunnelling^17^,^18^. In other words, the inversion layer increased the availability of states at the valence band, resulting in an enhanced tunnelling probability in the HCAP. Since the measured tunnelling current of 0.8 A cm^−2^ at 1 V falls on the trend of previously benchmarked metal/HfO_2_/n-Si MOS structures^19^, this implies that the current for both the NHCAP and HCAP is a result of direct tunnelling mechanism.

### High performing tunnelling-based thin-film transistor

**Figure 3:**
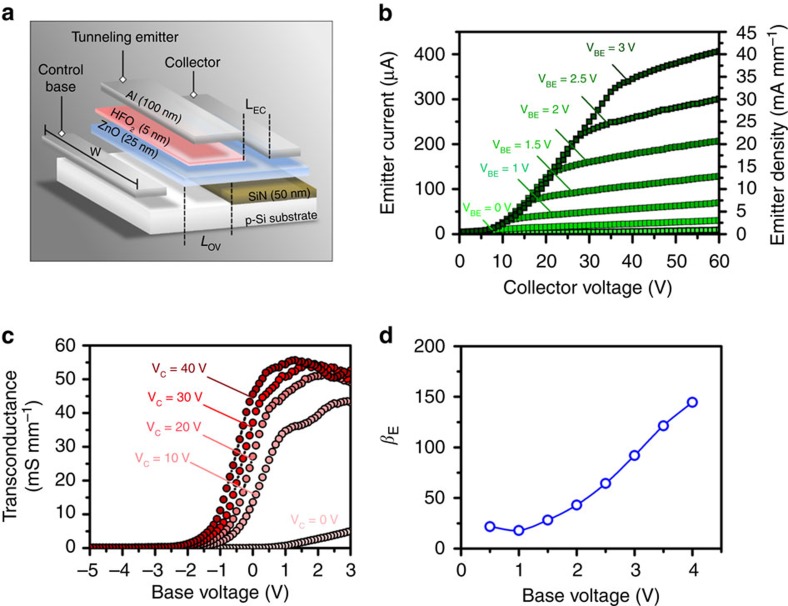
DC characteristics as a three terminal device. (**a**) The cross-section of the TJT based on the HCAP; (**b**) the measured family curves in common-emitter mode from *V*_BE_=0 to 3 V; (**c**) the transconductance of the TJT in the common-emitter mode; and (**d**) the forward current gain between emitter and base current (*β*_E_).

The utility of the enhanced tunnelling current associated with the inversion p-type layer in n-ZnO was also explored. A third, electron collector terminal, was added to create a tunnelling transistor device based on the HfO_2_/n-ZnO/p-Si junction MOSCAP, or in short, a bipolar tunnelling junction transistor (TJT). Figure 3a shows the layers and cross-section of the TJT. Silicon nitride (SiN) was used to isolate p-Si from n-ZnO where no contact was desired.

To observe transistor action, the n-ZnO/p-Si/HfO_2_ TJT was treated as a bipolar junction transistor (BJT) and modulated via the p-Si, or base, with the applied potential *V*_BE_. This in turn modulated by the inversion layer or ‘referred base'. The base-, emitter- and collector-width W was 10 μm, the Si/ZnO pn-junction overlap *L*_OV_ was 5 μm, and the emitter to collector length *L*_EC_ was 10 μm. When the collector terminal was positively biased, electrons that tunnelled from the emitter metal, through the HfO_2_ barrier and into ZnO were swept to the collector by the lateral electric field. Furthermore, since the device can be modelled as a BJT described in a phenomenological Ebers–Moll model, the transconductance directly stems from the change in the tunnelling current with respect to the base-emitter voltage.

A fraction of the electrons released from the emitter recombined with the holes in the inversion layers and manifested as parasitic losses, however, the majority of the electrons were eventually injected to the collector region. The current saturated as a direct result of a pinched-off virtual p-n junction between the p-type inversion layer and the n-type collector, giving rise to a characteristic transistor action. In Fig. 3b, the current–voltage family of curves are shown. In taking advantage of the p-inversion layer induced enhanced tunnelling mechanism, current density at the emitter was able to reach as high as 45 mA mm^−1^ at just 3 V base voltage. Despite the high current density, no electrical degradation was observed under these power conditions in the device, even up to a collector voltage of 60 V. A clear difference in the OFF-state and ON-state transconductance was also measured in Fig. 3c, reaching as high as 50 mS mm^-1^ and exhibited near ideal operational unidirectionality. To the best of our knowledge, it is the highest current and transconductance density for any TFT device to date (Table 1)^20^,^21^,^22^,^23^,^24^,^25^,^26^.

In Fig. 3d, forward differential emitter current gain *β*_E_ between the base and emitter current was evaluated and revealed that, despite parasitic losses to the base, the emitted tunnelling electrons were collected at a greater rate than the base leakages. Quantitatively, *β*_E_ increased in efficiency as base voltages increased, eventually exceeding 100 when greater than 3 V with an upwards trend. The gain mechanism is analogous to the principles outlined in Ebers–Moll configuration for BJTs, with the base-collector element corresponding to the inversion-collector region, and the emitter-base element corresponding to the metal-oxide-inverted ZnO region. Unique to this architecture is that as higher fields are applied across the barrier between the emitter-base junction, the tunnelling efficiency increases, resulting in a continually improving current gain. The significant demonstrated gain is promising in the future utilization of selectively deposited p-type material to eliminate losses due to substrate leakages.

## Discussion

Comparing the two cases, it was evident that an inversion layer was able to form and sustain itself in an HfO_2_/n-ZnO/p-Si structure, and with it, an ability to control both carrier species. In contrast to the HfO_2_/n-ZnO only MOSCAP, voltage dependence of charge was eventually lost during accumulation operating voltages (positive biases) but was not hindered during inversion (negative biases). These observations are also not limited to ZnO. A strong inversion layer will form, so long as the semiconductor meets two important criteria: a supply of minority carriers, and the diffusion length, is respectably larger than the thickness of the wide-bandgap film itself. In order to determine the practicality of a wide-bandgap inversion layer, we also realized a unique transistor which took advantage of the enhanced tunnelling current associated with the formation of this inversion layer coupled with the ultrathin barrier dielectric. In conclusion, we have demonstrated an inversion layer within an n-type wide-bandgap semiconductor ZnO. The inversion layer was practically realized as a thin-film bipolar HfO_2_/n-ZnO/p-Si TJT operating solely at the inversion regime. Without an inversion layer, the tunnelling current density at similar biasing conditions were smaller by almost two orders. It is important to note that further improvements of the device characteristics can be made by controlling the leakage and parasitic resistances associated with the collector and base. Materials other than silicon could be explored for the control base to create the inversion layer referred base in the wide-bandgap material. The use of this sustainable wide-band gap inversion layer resulted in a transistor type that is unmatched in output performance by all existing TFT devices known. The potential of a wide-bandgap inversion layer lays foundations for a field of wide-bandgap thin-film high-performance devices that can now be explored.

## Methods

### ZnO and HfO_2_ growth

On a p-Si substrate, 25 nm of ZnO was deposited via atomic layer deposition (ALD) at 130 °C. This was followed by 30 cycles of ALD (PEALD) of HfO_2_ at a temperature of 100 °C, respectively. The ALD of ZnO and HfO_2_ were done in a Kurt J. Lester ALD150-LX reactor. Argon was used as both the carrier and the purge gas. The ZnO layer was deposited by a sequence of 0.03 s diethylzinc (DEZ) pulse—5.00 s Ar purge—0.05 s water (H_2_O) pulse—10.00 s Ar purge at 130 °C. The HfO_2_ was deposited using a plasma-enhanced ALD (PEALD) process at 100 °C. Each PEALD cycle consisted of a sequence of 0.04 s tetrakis (dimethylamidohafnium) (TDMAH) pulse—5.00 s Ar purge—2.00 s remote oxygen plasma exposure- 2.00 s Ar purge. The growth per cycles (GPCs) of ZnO and HfO_2_ were 1.74±0.01 Å per cycle and 1.33±0.01 Å per cycle, respectively. These values were measured by spectroscopic ellipsometry technique. The spectroscopic ellipsometry was performed using a J.A. Woollom M2000-DI ellipsometer.

## Additional information

**How to cite this article:** Shoute, G. *et al*. Sustained hole inversion layer in a wide-bandgap metal-oxide semiconductor with enhanced tunnel current. *Nat. Commun.* 7:10632 doi: 10.1038/ncomms10632 (2016).

## Figures and Tables

**Table 1:** Comparison of other state-of-the-art inorganic TFTs.

Device name	Year	Material	J* (mA mm^−1^)	*G*_M_ (mS mm^−1^)
Lan and Peng^20^	2011	IGZO TFT	21.3	1.2
Li *et al*.^21^	2013	ZnO TFT	6.25	1.67
Carcia *et al*.^23^	2006	ZnO TFT	16	31.1
Barquinha *et al*.^22^	2006	IGZO TFT	15.7	14.3
Li *et al*.^24^	2014	Black P TFT	1.18	25
Radisavljevic *et al*.^25^	2011	MoS_2_ TFT	0.2	18.8
Lee *et al*.^26^	2014	IGZO TFT	29.2	33.3
Current work	2015	ZnO TJT	63	56

TFTs, thin-film transistors; TJT, tunnelling junction transistor.

^*^Current was evaluated at highest drain/collector voltage at highest or comparable gate/base voltage and normalized to the gate/base width.
